# Factors influencing employers’ support for employees with acquired brain injuries or mental illness to return to- and stay in work: A qualitative systematic review

**DOI:** 10.3233/WOR-230214

**Published:** 2024-09-11

**Authors:** Kristelle Craven, Blanca De Dios Pérez, Jain Holmes, Rebecca Fisher, Kathryn A Radford

**Affiliations:** a Centre for Rehabilitation & Ageing Research (CRAR), University of Nottingham, Nottingham, UK; b Medical Directorate, NHS England, UK

**Keywords:** Return to work, vocational rehabilitation, employment, work, work engagement, systematic review

## Abstract

**BACKGROUND:**

People with acquired brain injuries (ABIs) often experience residual limitations and co-morbid mental illnesses that restrict work participation. Employers are key in enabling successful return-to-work and job retention.

**OBJECTIVE:**

This review aimed to explore employers’ perspectives of factors influencing their support for people with ABIs and/or mental illness to return to- and stay in work. Review questions focused on barriers and facilitators to their support, and contextual characteristics present at the time.

**METHODS:**

Five databases were searched from October 2010 until November 2023 for relevant qualitative studies published in English. Findings from included studies (*N* = 25) were synthesised using thematic synthesis.

**RESULTS:**

Included studies focused on employees with ABI or mental illness, rather than dually diagnosed ABI and mental illness. Employers’ support was influenced by their awareness/knowledge of- and attitudes towards the employee’s condition/illness; their skills and experience in supportive strategies; factors related to provision of work accommodations; and stakeholder influence. Similarities and differences in influential factors were observed across the ABI and mental illness literature. Contextual characteristics related to organisational characteristics, cultural taboo, and involvement of certain stakeholders.

**CONCLUSIONS:**

ABI survivors (with and without co-morbid mental illness) and their employers may benefit from specialist support and resources to guide them through the return-to-work process. Further research is needed to investigate employers’ knowledge of ABI and mental illness and supportive strategies. Exploration of the influence of other stakeholders, socio-demographic characteristics, and contextual factors on employers’ return-to-work and retention support for ABI survivors with co-morbid mental illness is warranted.

## 1 Introduction

Acquired brain injuries (ABI) are defined as any injury to the brain taking place after birth, with common causes including trauma, vascular accident, infection, cerebral anoxia, inflammation, or metabolic/toxic issues [1, 2]. Individuals with these injuries are often left with physical, communicative, cognitive, behavioural, and emotional impairments that restrict their ability to participate in a range of activities and roles, including work [2]. They may experience loss of independence and friendships, unemployment, and financial hardship [3, 4]. These losses, in turn, can be compounded by family members needing to care for the individual and losing- or having their employment jeopardised [4]. ABI survivors are also at increased risk for subsequently developing mental illnesses such as anxiety, depression, bipolar disorder, and schizophrenia [5, 6] and these illnesses may still be present years following an ABI [7–9]. Mental illness is invisible in nature and often undiagnosed [10], meaning its prevalence among ABI survivors may be even greater than research suggests. The costs of ABI and mental illness to the United Kingdom’s (UK) economy have been estimated at £15 billion [3] and £117.9 billion [11] a year respectively, and these have largely been attributed to lost work contributions. In a systematic review, strong evidence has shown that co-morbid mental illnesses are negatively associated with return-to-work (RTW) rates among ABI survivors [10]. A bi-directional relationship has been suggested, whereby poor functional abilities post-ABI increase the risk of developing psychiatric disorders; and 2) psychiatric disorders influence re-integration (thus negatively influencing recovery of function) [10]. The interplay between post-ABI function and mental health suggests a more complex RTW process with more challenges, and a greater level of support needed compared with an ABI survivor without co-morbid mental illness. Among this population sub-group, a lack of expertise and support to enable return to work has been reported [4]. Employers of these individuals may be required to liaise with a greater number of stakeholders across different teams and organisations, spend more time learning about the employee’s morbidities, and require greater skills in creativity and problem-solving. It is possible workplace resources (e.g., time and availability of the employer, training opportunities) may reduce employers’ opportunity to provide adequate RTW/retention support.

Workplace context also influences whether or not ABI survivors return to- and stay in work. For example, factors influencing job retention rates among ABI survivors include the type of work (e.g., manual versus non-manual), organisation size, their occupational role (e.g., manager versus non-manager), and workload [12]. Additionally, high workloads and inadequate general support and expertise, work accommodations and environments, workplace policy, and employer knowledge are RTW barriers among ABI survivors [13–15], individuals with mental illnesses [16], and those with co-morbid ABI and mental illness [4]. Facilitators for RTW and retention across these groups include appropriate work accommodations [16, 17], gradual RTW (e.g., gradual increases in working hours, responsibilities and/or workloads) [17–19], and supportive, collaborative relationships with co-workers and employers [16–18, 20–23]. Among stroke survivors, level of perceived employer support has been statistically significantly associated with RTW [24]. Employers are thus key in enabling successful RTW and retention of individuals with these conditions; and the importance of their role is recognised by national legislation [25], clinical guidelines [26], and the United Kingdom (UK) government [27].

Investigation as to how employers can be supported in the RTW process has been recommended [16] but prior to this, clearer understanding of employers’ experiences providing support for RTW and job retention is required. To date, no qualitative studies seem to have been conducted exploring employers’ perspectives providing RTW or retention support to people with dual diagnoses of ABI and mental illness. Therefore it was anticipated that a qualitative review on these types of studies would result in an empty review. Systematic reviews focusing on depression [28] or a stroke [17] have revealed various factors perceived by employers as being influential on work participation of employees. These include treatment and support from health professionals, communication style, and appropriate adjustment of workload and tasks. However, these findings were based on only a small number of studies including employer perspectives relating to stroke (*n* = 2) or depression (*n* = 3), and it is unclear whether these findings are transferable to employers of people with other mental illnesses or ABIs. It does not appear as though a systematic review has ever focused on ABI and mental illness side-by-side. A dual focus such as this may elucidate the wider array of factors potentially experienced when employers support ABI survivors with co-morbid mental illness to return to and stay in work. Given the negative impact of co-morbid mental illness on the RTW rates of ABI survivors [10], increased understanding of what an employer might experience in these circumstances is important. For example, it may lead to future interventions aimed at improving employer support to be designed in a way that makes them more contextually relevant, useful, and feasible in real-life settings. Such knowledge and understanding may also help other stakeholders (e.g., health professionals) involved in the RTW and retention of people with ABIs and mental illness to be aware of the challenges potentially faced by employers; and work with them to overcome those challenges. Optimising employer support may lead to more ABI survivors with co-morbid mental illness successfully returning to- and retaining working roles, leading to benefits for ABI survivors and their families, their employers, organisations, and the UK economy. Thus, this review aimed to explore factors influencing employers’ support for employees to return to- and stay in work following ABIs or mental illness. Review questions were: 1) What barriers and facilitators have employers experienced when supporting employees with ABIs or mental illness to return to- and stay in work?; and 2) What contextual characteristics were present when these barriers and facilitators took place?

## 2 Methods

The Enhancing Transparency in Reporting the Synthesis of Qualitative Research (ENTREQ) statement was used to guide the structure and content of this article [29]. As this study was a systematic review, it was exempt from ethics committee approval.

### 2.1 Eligibility criteria

Qualitative studies exploring employer participants’ perspectives on factors influencing their support for employees to return to- and stay in work after an ABI and/or mental illness were eligible for inclusion. ABIs were defined as any injury taking place to the brain after birth [30]. Thus, ABI survivor employees may have suffered a stroke, traumatic brain injury (TBI), or other injuries related to an aneurysm, tumour, carbon monoxide poisoning, encephalitis, hypoxia/anoxia, and meningitis. Mental illnesses were not pre-defined to avoid missing studies where they had been included as an alternative umbrella term with other conditions or illnesses, e.g., episodic disability. In accordance with previous research involving employers [31], employers were defined as adults in senior occupational roles, such as supervisors, managers, or staff working within human resources (HR) or occupational health (OH) services or departments. Findings needed to have been reported in textual, non-numerical form to enable inclusion within a qualitative data synthesis. Studies reporting on the context of hiring disabled employees, rather than the RTW or job retention processes were excluded, as were those reporting in the context of an Individual Placement Support model (i.e., a work-focused health intervention incorporating work placements with job searching skills and one-to-one mentoring) [32]. These exclusion criteria were necessary to narrow focus of the review findings to employees already in employment at the time of their ABI and/or mental illness.

### 2.2 Information sources

A pre-planned search of five databases (OVID: MEDLINE, EMBASE, PsycINFO, ESBCO Host: CINAHL Plus with full text, Business Source Premier) was conducted by KC for articles published in English from October 2010 until August 2022. Databases were selected according to relevance of their content to the review aim, via discussion with the review team and an expert in systematic review searches. An update search was completed from August 2022 until November 2023. To keep the number of included studies manageable within the review timeframe, the number of databases searched was limited to five, the start date of 2010 was selected, and grey literature and books were excluded. Where possible, searches were limited to studies of human participants in adult age ranges. Reference lists of included studies were hand searched, and authors of conference abstracts were contacted to locate further studies.

The electronic search strategy was constructed by KC using relevant search terms related to the following: employers; return to-/stay in work; qualitative. No condition-related terms were used to avoid missing relevant studies focusing on general sick leave or disability management (e.g., that might include employers of people with mental illness or ABI).

### 2.3 Study screening and selection

**Fig. 1:**
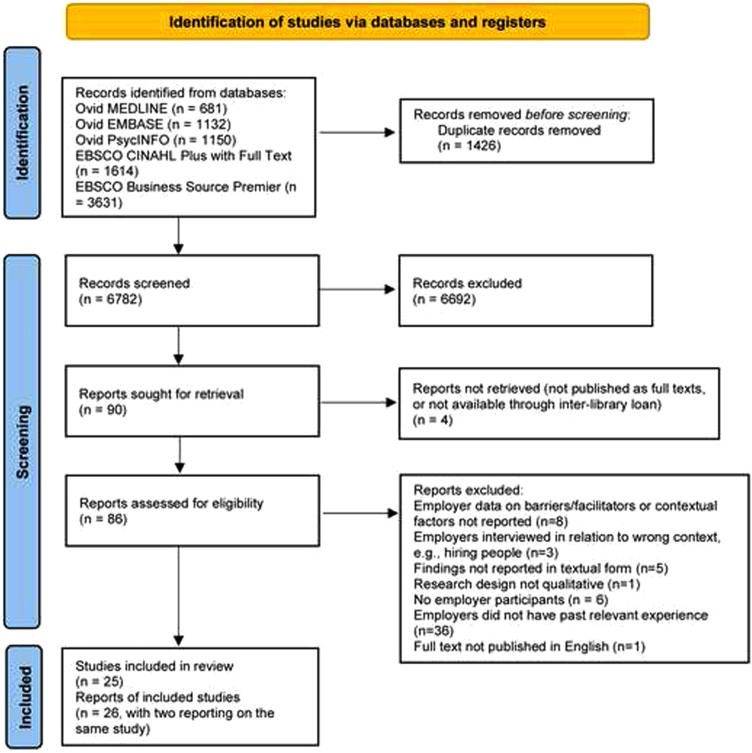
PRISMA flow diagram.

KC screened titles/abstracts using Endnote (version X9) [33]. Potentially eligible full texts were screened by KC; full texts marked as “include” or “unsure” were screened independently by BD or CS. Uncertainties or disagreements were resolved through discussion. Further details of the study selection process are presented in a Preferred Reporting Items for Systematic Reviews and Meta-analyses (PRISMA) flow diagram [34] (Fig. 1).

### 2.4 Data extraction and quality appraisal

Study characteristics data were extracted by KC using a data extraction form, adapted from a template from Cochrane Effective Practice and Organisation of Care (EPOC) [35]. To enable collection of data on context, the form included the country in which the study was conducted, health conditions of employees and reasons for employer support (e.g., RTW or job retention), occupational roles/responsibilities of the employer, organisation size and type, details of relevant country legislation and employer obligations, and set-up of RTW/retention support (e.g., support typically available through the public healthcare system). No further data extraction was required because the thematic synthesis was carried out within NVivo (version 12) software [36].

The quality of included studies was assessed using the Critical Appraisal Skills Programme (CASP) Qualitative Checklist [37]. This tool involves appraisal of the validity of study results, how the results were obtained, and whether the results are valuable [37]. It is commonly used in health-related qualitive reviews of evidence; and its usage is endorsed by the Cochrane Qualitative and Implementation Methods Group [38]. KC and BD independently assessed quality; discrepancies were resolved through discussion.

### 2.5 Thematic synthesis

KC applied the Review question-Epistemology-Time/Timescale-Resources-Expertise-Audience and purpose (RETREAT) framework [39] to inform the decision to employ thematic synthesis [40] as the synthesis methodology, and this decision was checked with the review team. Use of the framework enables identification of the synthesis methodology most appropriate for the review being conducted, based on the review question, the timeframe and financial/physical resources for conducting the review, knowledge/skill of the reviewers, anticipated reader expectations and intended use of findings, and the type of data available to address the review question. To initiate the synthesis, KC familiarised herself with the data before completing line-by-line coding from results and discussion sections within included full texts, using NVivo (version 12) software [36]. An inductive approach was taken during the coding stage to ensure thorough exploration of the employers’ perspectives. To increase understanding of barriers and facilitators to employer support, the Sherbrooke Model [41] was used as a sensitising framework to map them to the systems in which they took place (e.g., workplace system, healthcare system, etc). KC compared and organised codes into 22 descriptive themes and summarised them with example quotes. BD independently checked the summary against the data and suggested changes to theme construction. KC examined and interpreted the descriptive themes to generate overarching analytical themes for the barriers and facilitators experienced by employers (Research question 1). Data concerning contextual factors were included alongside barrier/facilitator data to enhance understanding of the contexts in which the barriers and facilitators took place (Research question 2). Analytical themes were reviewed by BD and JP, and changes made via group discussion.

## 3 Results

Characteristics of the 25 included studies are presented in Table 1. None of the studies included employers of ABI survivors with co-morbid mental illness, so findings related only to employees with ABI or mental illness (i.e., singular morbidities). Most were conducted in Sweden [31, 42–47], Canada [48–52], or the UK [53–57]; with others conducted in the USA [58, 59], Barbados [60], Denmark [61], the Netherlands [13], New Zealand [62], Australia [63], and South Africa [64]. Most were published in 2016 or later (*n* = 20), and seven interviewed employers following participation in a vocational rehabilitation intervention [44, 46, 49, 51, 55, 63, 64]. Employers’ occupational roles were commonly reported as supervisor/manager, HR staff, OH nurse, small business owners, director, or coordinator. Only ten studies reported on organisation size; using criteria employed by the UK Government [65], these were classified as including employers from a mix of micro- (0–9 employees) small- (10–49 employees), medium- (50–249 employees), and/or large-sized organisations (≥ 250 employees) [13, 45, 46, 48, 49, 53, 56, 59, 61].

**Table 1:** Characteristics of included studies (N = 25)

First author, (Year of publication) and country	Study aim/research questions	Study design, data collection method	Details of linked intervention (if applicable)	Employer participant characteristics		Size and type of organisational setting	Health condition/s of employees supported by employers	Contextual reason/s for employer support (e.g., work retention)
Sample size, Gender, Age, Race/ethnicity	Occupational role/s and responsibilities
Bush, 2016 [58] USA	To explore how adults with TBI and the people associated with them describe employment experiences post-injury	Multiple case study Semi-structured interviews	Not applicable	*N* = 1 Female: *n* = 1 Age and race/ethnicity not reported	Job supervisor	Crop insurance agency. No further details reported	Severe TBI	Post-injury RTW
Coole, 2013 [53] UK	To explore perceptions and experiences of employer stakeholders in supporting employees to RTW post-stroke, identify key aspects linked to successful RTW, and obtain their views regarding a VR RTW service	Qualitative study Semi-structured interviews	Not applicable	*N* = 18 Gender, age and race/ethnicity not reported	Human resources staff (*n* = 3), occupational health physician (*n* = 1), occupational health nurse (*n* = 3), small business owners (*n* = 3), a managing director (*n* = 1), a manager (*n* = 1), line manager/supervisors (*n* = 3), and a disability employment advisor (*n* = 1)	Organisations in service (*n* = 12), manufacturing (*n* = 2), engineering (*n* = 3) or various industries (*n* = 1). Based in private (*n* = 10), public (*n* = 5), or voluntary sectors (*n* = 3). Most organisations were large (> 250 employees: *n* = 8); others were micro- (< 10 employees: *n* = 4), small- (10–50 employees; *n* = 1), or medium-sized (>50–250; *n* = 3)	Stroke	Post-stroke RTW
Devonish, 2017 [60] Barbados	Research questions related to managerial definitions and views of mental health and illness in the workplace, their experiences with people with mental illness, and perceived support/resources needed to manage and support employees with mental illness within the workplace	Explorative qualitative research design Two focus groups (one for public sector managers, one for private sector managers)	Not applicable	*N* = 16 Male: *n* = 8 Female: *n* = 8 Age range: 32–59 years Race/ethnicity not reported	Public sector managers (*n* = 8): included supervisory and/or managerial job roles, e.g., senior executive/accounting/administrative officers, and a sergeant from the local police force Private sector managers (*n* = 8): included front line supervisors, and HR and operations managers.	Public sector managers worked in the civil service Private sector managers worked in personal and health services, finance, tourism and hospitality, construction, and retail/wholesale industries	Mental illness	General support for employees with mental illness to cope with their condition within the workplace
Donker-Cools, 2018 [15] Netherlands	To investigate which factors provide solutions to RTW problems, or hinder or facilitate RTW as experienced by patients with ABIs and employers	Explorative qualitative study Semi-structured interviews	Not applicable	*N* = 7 Male: *n* = 4 Female: *n* = 3 Middle-aged. Race/ethnicity reported	Supervisor (*n* = 1), line manager (*n* = 3), HR manager (*n* = 2), director (*n* = 1)	Organisational settings included a town hall (1900 employees), an academic hospital (11,000 employees), a national sports federation (29 employees), a police office (1230 employees), a factory (240 employees), and two schools (2965 and 140 employees)	Non-progressive ABI	Post-injury RTW
Gignac, 2021 [48] Canada	To increase understanding of employer representatives’ perspectives on disability communication-support processes	Explorative qualitative study Semi-structured interviews	Not applicable	*N* = 27 Male: *n* = 7 Female: *n* = 20 Age and race/ethnicity not reported	Supervisor/manager (*n* = 4), disability manager (*n* = 7), HR personnel (*n* = 5), worker advocates/union representatives (*n* = 5), labour lawyers representing workers, a large union or large organisation (*n* = 3), medical director and OH nurse (*n* = 2), health and safety representative (*n* = 1). Also included 5 employer representatives with lived experience of physical or mental episodic disability	Small (< 100 employees) = 6, medium or large (≥100 employees) = 21 Organisations were based in business, finance and professional services (*n* = 4), education or government (*n* = 6), healthcare (*n* = 6), manufacturing, construction or utilities (*n* = 4), non-profit (*n* = 1), service or retail (*n* = 1), or multiple sectors (*n* = 5)	Episodic disabilities (e.g., depression, anxiety, arthritis)	Work retention of employees with episodic disabilities
Gordon, 2015 [62] New Zealand	To investigate the factors critical in enabling and sustaining open employment of mental health service users, from perspectives of employees and their employees	Multiple case study Semi-structured interviews	Not applicable	*N* = 14 Male: *n* = 4 Female: *n* = 10 Age and race/ethnicity not reported	Occupational role/responsibilities not reported	Private sector (*n* = 7; e.g., small owner operated bakery, electrical retailer, pharmacy, very large supermarket) Public sector (*n* = 4, e.g., school, university, police force) Non-governmental organisations based in mental health sector (*n* = 3)	Mental illness (five employees also had co-morbid physical illnesses or disabilities - no further details reported)	General management and support for employees with mental illness in open employment
Gouin, 2019 [49] Canada	To explore influence of decision-making processes on the RTW of employees with common mental disorders or musculoskeletal conditions	Secondary analysis of three multiple case studies Semi-structured interviews	Interdisciplinary work rehabilitation intervention with content relating to reassurance, avoidance behaviour, reduction of fears, collaboration between stakeholders and a progressive RTW	*N* = 19 Gender, age and race/ethnicity not reported	Immediate supervisors (*n* = 14) and human resources managers (*n* = 5)	Those who supported employees with mental illness were based in the service sector; within a government organisation and a large private organisation (> 500 employees)	Common mental disorders or musculoskeletal conditions	RTW due to mental illness or musculoskeletal condition
Hellman, 2016 [42] Sweden	To describe and explore stakeholders’ views of important aspects of the RTW process for stroke survivors, and explore how their contrasting perspectives may influence RTW services	Exploratory qualitative study Focus groups	Not applicable	*N* = 5 Male: *n* = 3 Female: *n* = 2 Age and race/ethnicity not reported	Not reported	Not reported	Stroke (occurred 7–18 years prior to study)	Post-stroke RTW
Holmlund, 2022a [43] Sweden	To identify ethical issues arising during RTW coordination for employees with common mental disorders	Descriptive qualitative study Semi-structured interviews	Not applicable	*N* = 10 Male: *n* = 2 Female: *n* = 8 Age and race/ethnicity not reported	Coordinator (*n* = 2), OHS nurse (*n* = 2), CEO (*n* = 2), HR personnel (*n* = 4)	Details not reported	Mild-to-moderate depression, adjustment disorder, or anxiety	RTW due to mental illness
Holmlund, 2022b [44] Sweden	To explore employee and managerial perceptions of reasons for sick leave resulting from common mental disorders, using a transactional perspective of gender norms and everyday life occupation	Exploratory qualitative study Semi-structured interviews	Intervention offered as one arm of an RCT. Aimed to improve RTW process of participant. Coordinator supported employees and employers to collaboratively identify RTW issues and come up with solutions to issues	*N* = 11 Male: *n* = 4 Mean age (years) (range): 49 (36–63) Female: *n* = 7 Mean age (years) (range): 44 (32–54) Race/ethnicity not reported	First-line managers (*n* = 7), chief executive officer (*n* = 1), school principal (*n* = 1). Details of other two managers’ roles not reported. All were responsible for rehabilitation of a participant included in the linked RCT	Private sector (*n* = 7), municipality or regional sector (*n* = 4)	Mild-to-moderate depression, adjustment disorder, or anxiety	Work retention of employees, just prior to them being absent due to mental illness
Irvine, 2023 [56] UK	To explore how small business contexts influence support and management of mental health problems in work environments	Exploratory qualitative study Semi-structured/narrative interviews	Not applicable	*N* = 21 Male: *n* = 4 Female: *n* = 17 Age and race/ethnicity not reported	Managers (*N* = 21)	Small businesses of 50 or less employees, in charity (*n* = 7) or private sectors (*n* = 14). Industries included social care (*n* = 3), healthcare (*n* = 4), skilled manual (*n* = 1), manufacturing/sales (*n* = 1), consultancy (*n* = 3), law (*n* = 1), community development (*n* = 3), construction (*n* = 1), digital marketing (*n* = 1), food production/retail (*n* = 1), animal care (*n* = 1), and information and advice (*n* = 1).	Mental health problems (e.g., anxiety, depression, or stress)	Work retention of employees with mental health problems
Lemieux, 2011 [50] Canada	To record supervisors’ perceptions of factors hindering or facilitating RTWs of employees with common mental disorders	Exploratory qualitative study Semi-structured interviews	Not applicable	*N* = 11 Male: *n* = 8 Female: *n* = 3 Age and race/ethnicity not reported	Supervisors with experience in RTW of employees absent due to common mental disorders.	Medium (*n* = 4) or large-sized companies (*n* = 7) in education (*n* = 4), financial (*n* = 3), food retail (*n* = 1), transportation (*n* = 1), public service (*n* = 1) and health (*n* = 1) sectors	Common mental disorders	RTW due to mental illness
Lexén, 2019 [45] Sweden	To develop a model to explain how attitudes, knowledge and experiences of employers and rehabilitation professionals influence strategies utilised during RTW of employees with mental illness	Grounded theory Interviews (type not reported)	Not applicable	*N* = 23 Male: *n* = 9 Female: *n* = 14 Mean age (years) = 51.8 Race/ethnicity not reported	Details of occupational roles/responsibilities not reported	Manufacturing (*n* = 3); pedagogic work (*n* = 4); installation, operation and maintenance (*n* = 1); healthcare (*n* = 5); hotel/restaurant (*n* = 1); sales, purchasing and marketing (*n* = 2); information technology (*n* = 3); construction (*n* = 2); administration, economy and law (*n* = 1); and police (*n* = 1). Based in private (*n* = 14), public (*n* = 6), and governmental (*n* = 3) sectors, with numbers of employees including < 5 (*n* = 6), 5–10 (*n* = 8), and > 50 (*n* = 10)	Mental illness	RTW due to mental illness
Libeson, 2021 [63] Australia	To understand experiences of employers of TBI survivors who have received comprehensive VR, what is involved in supporting these employees, and the needs of the employers themselves	Explorative qualitative study Semi-structured interviews	State-run VR program led by TBI-specialist VR occupational therapist (OT). Included work-site assessments, employer liaison, cognitive strategies, tailored work modifications, and ongoing support and monitoring in the workplace	*N* = 12 Male: *n* = 6 Female: *n* = 6 Age range (years): 30–70 Race/ethnicity not reported	Direct manager (*n* = 8); RTW/HR coordinator (*n* = 2); Director and direct manager (*n* = 2)	Small-sized organisations (*n* = 2; private entertainment, private public relations); medium-sized organisation (*n* = 1, public hospital); large-sized organisations (*n* = 9, private finance, public service/government, private retail, private hospital, public service/construction, private hospitality)	TBI	Post-TBI RTW
Marois, 2020 [51] Canada	To evaluate the feasibility of a RTW program for employees with common mental disorders, from the perspectives of employers, insurers, employees and unions	Sequential mixed-methods design Group discussion	Adapted Therapeutic Return-to-Work (TRW) Program aimed to facilitate RTW of employees with common mental disorders. Included Work Disability Diagnosis Interview; preparation; therapeutic RTW; coaching to develop employee work capacity; and maintenance support	*N* = 7 Female: *n* = 7 Median (range) in years: 37 (29–60) Race/ethnicity not reported	Employers worked in a health office (*n* = 5) or in HR (*n* = 2)	No details of organisational settings reported	Common mental disorders	RTW following sick leave of ≥ 6 months due to mental illness
Morant, 2021 [54] UK	To explore experiences and views of employees with mental health problems, mental health clinicians, and managers of social firms, on the value of social firms for VR, wellbeing and employment of individuals with mental health problems	Explorative qualitative study Semi-structured interviews, focus group	Not applicable	*N* = 12 Details on age and race/ethnicity not reported	Managers of social firms, where at least one employee had a mental health problem	Social firms were mostly small (average number of people employed = 7), all based in England. Range of sectors including training (*n* = 2); recycling (*n* = 2); and one each of gardening, printing, market research, health foods, framing, textiles, and travel agent	Mental health problems	Work retention of employees with mental health problems within social firms
Nielsen, 2023 [57] UK	To examine line managers’ supportive behaviours towards employees who had returned from work following long-term sickness absence due to common mental disorders	Longitudinal descriptive qualitative study Semi-structured interviews (managers interviewed up to three times if they were managing a returned worker at the time of the data collection)	Not applicable	*N* = 20 Male: *n* = 7 Female: *n* = 13 Age (years): 25–34: *n* = 1 35–44: *n* = 5 45–54: *n* = 7 55 or older: *n* = 4 Not reported: *n* = 3. Details on age/ethnicity not reported	Line managers	Sizes of organizations not reported. Managers worked in publishing (*n* = 1), information technology (*n* = 1), police and emergency services (*n* = 2), education and research (*n* = 2), administration (*n* = 8), and healthcare services (*n* = 6)	Common mental disorders (i.e., stress, anxiety, depression)	Work retention of employees with common mental disorders
Öst Nilsson, 2019 [46] Sweden	To describe and explore managerial and co-workers’ experiences of RTW processes involving a stroke survivor colleague who took part in a client-centred VR programme	Qualitative explorative design Two semi-structured interviews per employer: conducted ≤3 weeks after beginning of work trial, and then 8–9 weeks later	Person-centred, individually tailored VR intervention delivered by OTs. Employers received information regarding impact of stroke on work abilities, and met with OTs, stroke survivor employees (and social insurance officers) to plan and evaluate work trials	*N* = 4 Gender, age and race/ethnicity not reported	Managers who worked closely with the stroke survivor employees and had insight into their RTW process	Organisations in following sectors: Transport (*n* = 1; 100 employees); manufacturing (*n* = 2; 20–50 employees); and education (*n* = 1; 12 employees)	Mild or moderate stroke	Post-stroke RTW during a VR programme
Porter, 2019 [33] Sweden	To explore employers’ knowledge, beliefs, and strategies used to provide support for employees with mental illness	Grounded theory Interviews	Not applicable	*N* = 24 Male: *n* = 10 Female: *n* = 14 Mean (range) in years: 49.2 (39–62) Race/ethnicity not reported	Details of occupational roles/responsibilities not reported	Politics/government (*n* = 1); administration, economy and law (*n* = 1); police (*n* = 1); construction (*n* = 1); information technology (*n* = 2); sales, purchasing and marketing (*n* = 4); hotel or restaurant (*n* = 1); installation, operation and maintenance (*n* = 1); healthcare (*n* = 4); manufacturing (*n* = 3); and education (*n* = 5).	Mental illness	General management and support for employees with mental illness to cope with their condition within the workplace
Radford, 2018a [55] UK	Group 1: To identify the most valued intervention components in practice, from the perspectives of TBI survivors and employers Group 2: To identify the most important outcomes of VR, from the perspectives of TBI survivors, service providers, and employers	Part of mixed methods process evaluation nested within feasibility trial of a VR intervention Semi-structured interviews	Aim of the Early Specialist Traumatic brain injury Vocational Rehabilitation (ESTVR) intervention is to prevent job loss among employed TBI survivors. Individually tailored, delivered by OTs. Employers and family members are supported to increase their understanding of the impact of the injury on the individual and their work ability.	Group 1: *n* = 6 Group 2: *n* = 12 Gender, age, and race/ethnicity not reported	Group 1: Coordinator (*n* = 1); manager (*n* = 2); head of department (*n* = 1); staff member from occupational health service (*n* = 1); and an assistant director (*n* = 1) Group 2: Human Resource manager (*n* = 1); occupational health doctor (*n* = 1); occupational health nurse (*n* = 1); disability employment advisor (*n* = 1); line managers (*n* = 7); and a personal injury solicitor (*n* = 1)	Group 1: A recycling charity, a disability inclusion service, a Trust in the National Health Service, a restaurant, a university occupational health service, and a school. Group 2: Private occupational health companies (*n* = 2), manufacturing companies (*n* = 2), universities (*n* = 2), TBI charities (*n* = 3), a voluntary sector organisation (*n* = 1), a private solicitor (*n* = 1), and a government employment agency (i.e., JobCentrePlus) (*n* = 1).	TBI	Post-injury RTW and work retention
Santy 2016 [59] USA	To explore implications of the RTW transition for TBI survivors for policy, address the literature gap, and identify factors contributing to success of RTW programs in Washington State	Ethnographic study Semi-structured interviews	Not applicable	*N* = 6 Male: *n* = 3 Female: *n* = 3 Age range: 52–62 years Race/ethnicity not reported	Business owner (*n* = 1), director (*n* = 1), adjudicator (*n* = 1), consultant (*n* = 1) and a manager (*n* = 1)	Number of employees per organisation ranged from 12 to 75000 in the private sector (*n* = 2), and 3000 to 3200 in the public sector (*n* = 2). Total of 75 employees in one non-profit organisation	Mild to moderate TBI	Post-injury RTW and work retention
Soeker, 2019 [64] South Africa	To explore perceptions and experiences of employers and caregivers of individuals with TBI RTW after completing a VR program based on the Model of Occupational Self-Efficacy (Moose)	Exploratory qualitative study Semi-structured interviews	Four-stage VR intervention. Involved reflective processes, enhancement of individual capabilities, work simulation, and RTW for ≥4 months	*N* = 10 Gender, age and race/ethnicity not reported	Junior supervisor (*n* = 1), senior supervisor (*n* = 1), floor manager (*n* = 4), general manager (*n* = 2), manager (no other details reported) (*n* = 1), business owner (*n* = 1)	Food outlets (*n* = 6), a local beverage factory, a security company and a non-governmental organisation	Mild to moderate TBI	Post-injury RTW (linked to a VR intervention)
St-Arnaud, 2011 [52] Canada	To define the paradigms and practices of workplace stakeholders involved in managing and following up RTW of employees following sickness absence due to mental illness	Qualitative study Semi-structured interviews	Not applicable	*N* = 24 Gender, age and race/ethnicity not reported	Senior managers (*n* = 7): Responsibilities included surveying workforce in relation to organisational climate, and producing and disseminating absence statistical information Direct supervisors (*n* = 10): Responsibilities included supporting staff, and preventing and managing staff absence OH officers (*n* = 7): Responsibilities included medical and administrative follow-up of employees who received disability insurance, ensuring adequacy of treatment plans, and reviewing scheduled RTW dates	Participants recruited from 7 out of 11 departments in one workplace. This workplace had an in-house OH department	Mental illness	RTW due to mental illness
Thisted, 2020 [61] Denmark	To investigate employers’ attitudes for management of employees’ depression, with focus on the employers’ challenges and opportunities in providing support	Qualitative study Semi-structured interviews	Not applicable	*N* = 5 Male: *n* = 1 Female: *n* = 4 Age range (years): 45–72 Race/ethnicity not reported	Management positions, all with more than 5 years leadership experience	Private psychological care clinic (*n* = 1), and public sector organisations based in education (*n* = 2), healthcare (*n* = 1), and the social sector (*n* = 1) Organisations were small- (< 50 employees; *n* = 2) or medium- sized (50–250 employees; *n* = 3)	Depression	General management and support for employees with mental illness to cope with their condition within the workplace
Tjulin, 2010 [47] Sweden	To explore experiences of workplace actors’ social relations, and how work-based organisational dynamics in RTW extend before and after initial return of sick-listed employees	Grounded theory Interviews	Not applicable	*N* = 8 Male: *n* = 1 Female: *n* = 7 Age and race/ethnicity not reported	Supervisors (*n* = 6), HR managers (*n* = 2)	Seven work units within three public sector organisations	Of the 7 employees, four had been diagnosed with mental illnesses (two had co-morbid physical conditions); three others had musculoskeletal issues	RTW after illness (with sick leave lasting at least 1 month)

Nine studies included employers of employees with ABIs (e.g., traumatic brain injury, stroke) [13, 42, 46, 53, 55, 58, 59, 63, 64]. These employers’ organisations included private and public healthcare, charities, manufacturing, public service/government, retail, and higher education. These studies focused on RTW of employees, with two considering work retention also [55, 59].

Sixteen studies included employers of employees with mental illness, including depression, anxiety, and adjustment disorder [31, 43–45, 47–52, 54, 56, 57, 60–62]. These employers’ organisations included finance, business, information technology, manufacturing, tourism, hospitality, construction, retail, public service/government, administration, law, education and research, publishing, community development, digital marketing, food production, animal care, consultancy, social care, and healthcare. Seven studies focused on RTW of employees [43, 45, 47, 49–52]; the remainder focused on work retention [31, 44, 48, 54, 56, 57, 60–62].

### 3.1 Quality appraisal of the included studies

Quality appraisal ratings are presented in Table 2. All included studies clearly stated their research aims; and their choices of qualitative methodology, research designs, and data collection methods were deemed appropriate. Two studies reported insufficient detail to inform judgment on appropriateness of recruitment strategies [49, 60]; nineteen studies did not report consideration of the relationship between the researcher and participants [13, 31, 42–45, 47–54, 56, 58, 60, 61, 63]. Some studies reported insufficient detail to inform judgment on consideration of ethical issues (*n* = 2) [52, 56] and sufficiently rigorous data analysis (*n* = 3) [49, 56, 62]. Authors of one study [59] did not clearly state their findings. All other studies were judged as meeting these criteria. All included studies were deemed as having some value, e.g., by discussing their findings in relation to practice/policy or previous research, suggesting new areas for future research, and discussing how their findings could be applied in real life contexts. In studies conducted within specific contexts (e.g., a large organisation in Canada [52], social firms [54], and countries with very different health and social care systems, it was questionable how transferable their findings were outside of these contexts.

Weighting or exclusion of studies based on their quality appraisal was not conducted. The CASP tool was not designed with an accompanying scoring system, and it is suggested that ratings for actual domains are presented [66]. However, the developers suggest that if a “yes” rating cannot be assigned to the first three questions, then it may be considered poor-quality evidence [66]. As Table 2 shows, “yes” ratings were assigned to all studies on the first three questions, suggesting that no poor-quality evidence was included. Furthermore, weighting of individual studies would not have substantially influenced findings (i.e., there were other studies with “yes” ratings showing the same findings).

**Table 2:** Quality appraisal ratings for included studies (N = 25)

First author, (year of publication)	1. Was there a clear statement of the aims of the research?	2. Is a qualitative methodology appropriate?	3. Was the research design appropriate to address the aims of the research?	4. Was the recruitment strategy appropriate to the aims of the research?	5. Was the data collected in a way that addressed the research issue?	6. Has the relationship between researcher and participants been adequately considered?	7. Have ethical issues been taken into consideration?	8. Was the data analysis sufficiently rigorous?	9. Is there a clear statement of findings?
Bush (2016)	Yes	Yes	Yes	Yes	Yes	Can’t tell	Yes	Yes	Yes
Coole (2013)	Yes	Yes	Yes	Yes	Yes	Can’t tell	Yes	Yes	Yes
Gouin (2019)	Yes	Yes	Yes	Can’t tell	Yes	Can’t tell	Yes	Can’t tell	Yes
Lemieux (2011)	Yes	Yes	Yes	Yes	Yes	Can’t tell	Yes	Yes	Yes
Soeker (2019)	Yes	Yes	Yes	Yes	Yes	Yes	Yes	Yes	Yes
Donker-Cools (2018)	Yes	Yes	Yes	Yes	Yes	Can’t tell	Yes	Yes	Yes
Devonish (2017)	Yes	Yes	Yes	Can’t tell	Yes	Can’t tell	Yes	Yes	Yes
Gordon (2015)	Yes	Yes	Yes	Yes	Yes	Yes	Yes	Can’t tell	Yes
Hellman (2016)	Yes	Yes	Yes	Yes	Yes	Can’t tell	Yes	Yes	Yes
Ost Nilsson (2019)	Yes	Yes	Yes	Yes	Yes	Yes	Yes	Yes	Yes
Radford (2018)	Yes	Yes	Yes	Yes	Yes	Yes	Yes	Yes	Yes
Santy (2016)	Yes	Yes	Yes	Yes	Yes	Yes	Yes	Yes	Can’t tell
Lexén (2019)	Yes	Yes	Yes	Yes	Yes	Can’t tell	Yes	Yes	Yes
Marois (2020)	Yes	Yes	Yes	Yes	Yes	Can’t tell	Yes	Yes	Yes
Porter (2019)	Yes	Yes	Yes	Yes	Yes	Can’t tell	Yes	Yes	Yes
St-Arnaud (2011)	Yes	Yes	Yes	Yes	Yes	Can’t tell	Can’t tell	Yes	Yes
Thisted (2020)	Yes	Yes	Yes	Yes	Yes	Can’t tell	Yes	Yes	Yes
Tjulin (2010)	Yes	Yes	Yes	Yes	Yes	Can’t tell	Yes	Yes	Yes
Libeson (2021)	Yes	Yes	Yes	Yes	Yes	Can’t tell	Yes	Yes	Yes
Gignac (2021)	Yes	Yes	Yes	Yes	Yes	Can’t tell	Yes	Yes	Yes
Holmlund (2022a)	Yes	Yes	Yes	Yes	Yes	Can’t tell	Yes	Yes	Yes
Holmlund (2022b)	Yes	Yes	Yes	Yes	Yes	Can’t tell	Yes	Yes	Yes
Morant (2021)	Yes	Yes	Yes	Yes	Yes	Can’t tell	Yes	Yes	Yes
Irvine (2023)	Yes	Yes	Yes	Yes	Yes	Can’t tell	Can’t tell	Yes	Yes
Nielsen (2023)	Yes	Yes	Yes	Yes	Yes	Yes	Yes	Yes	Yes

### 3.2 Findings from the thematic synthesis

**Fig. 2:**
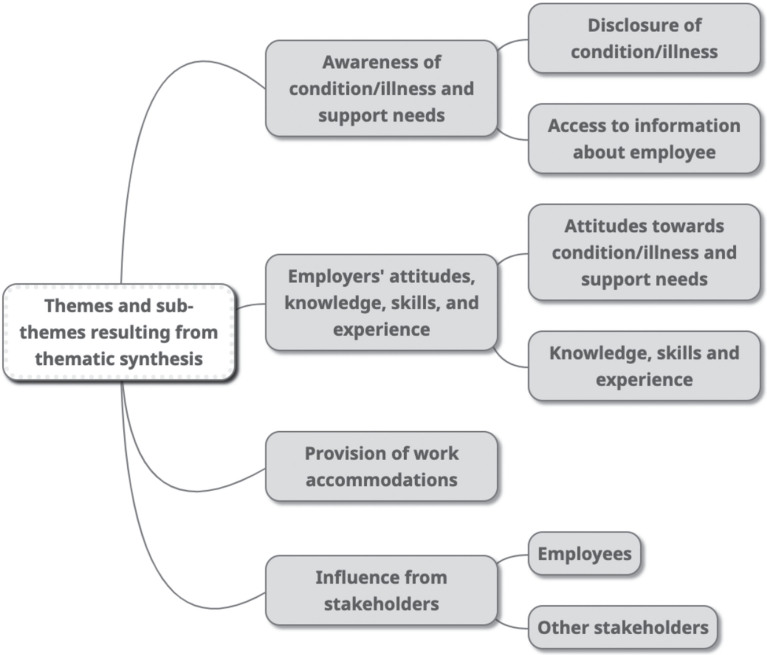
Themes and sub-themes from the thematic synthesis.

Themes that emerged from the thematic synthesis are presented in Fig. 2 and include: 1) Awareness of condition/illness and support needs; 2) Employers’ attitudes, knowledge, skills and experience; 3) Provision of work accommodations; and 4) Influence from stakeholders. Across all themes, barriers and facilitators to employer support took place throughout the RTW/retention process, relating to the employer themselves, the employee with the ABI or mental illness, and various environmental factors within the workplace, healthcare, legislative/insurance, and culture/politics systems. Direct quotes to illustrate the findings are presented in Table 3. The barriers and facilitators are summarised in Table 4, and reported within theme descriptions. Where reported, contextual characteristics surrounding the barriers and facilitators are described within the theme descriptions, and summarised in Table 5.

**Table 3:** Examples of study quotes per theme

Theme	Sub-theme	Example quote
Awareness of condition/illness and support needs	Disclosure of condition/illness
	Barriers:
	Employees described depression as something else, due to cultural taboo linked to depression	“ . . . depression is tabooed, and nobody talks about depression . . . In turn, employees may disclose their depression as stress or something else . . . ” [61] (Author interpretation)
	Employees with ABIs or mental illness not always aware of residual limitations and work-related challenges	[TBI survivor] “The worker is not usually knowledgeable until they step back into the work site, or once they get there and discover they can’t do some part of their work” [59]
		[Employees with episodic disabilities, e.g., depression, anxiety] “More commonly with a mental health condition, you’ve got subtler things: meltdowns, chronic lateness, inability to concentrate, disruptive behaviour, not fulfilling commitments, or not showing up for work regularly . . . We label them as complex cases, we try to be as good as we can. When somebody’s perception of their ability doesn’t match the reality, then we have to take those very delicately” [48]
	Facilitator:
	Disclosure of mental illness led to better employer understanding and supportive action	“When Pat^*^ did subsequently disclose his experience of mental illness, Shazza felt able to understand more fully and to offer support if required” (Author interpretation) [62]
	Access to information about employee
	Barriers:
	Employers omitted from disability support and RTW planning	[Employees with episodic disabilities, e.g., depression, anxiety] “At times, supervisors and workers were not included in discussions” (Author interpretation) [48]
	Lack of- or inadequate information from health professionals	[Employees with mental illness] “Employers also described a feeling of being “kept in the dark” when meeting with the different RTW services with regard to the employee’s rehabilitation. This made it difficult to provide adequate work accommodations” (Author interpretation) [45]
	Facilitators:
	Obtained information from employee (e.g., by asking them to get it in writing from health professional, or asking them to communicate their support needs)	[Stroke survivor employees] “Sometimes you can get the patient on your side and you can say, “Look, when you see your physio next, or whoever, can you ask them, can they put anything in writing?” and sometimes the physios will do that” [53]
Employers’ attitudes, knowledge, skills, and experience	Attitudes towards condition/illness and support needs
	Barriers:
	Employer support depended on whether they saw mental illness as a workplace or personal issue	“Opportunities to support employees with depression are influenced by whether depression is understood as a private matter that should be managed in the private sphere or embraced as a workplace issue that involves the responsibility of the employer” (Author interpretation) [61]
	“ . . . absences pertaining to mental illness versus absences pertaining to relational conflicts, disciplinary measures or problems related to personal life . . . some workers were given more support and more time to recover and had access to additional sessions under the employee assistance program (EAP). Other workers received telephone calls putting them under greater pressure, and were questioned and challenged regarding their treatment and health status” (Author interpretation) [52]
	Facilitator:
	Employees with ABIs or mental illness considered valuable for organizations	[TBI survivor employees] “I would characterize us as compassionate, and try to see the value of the individual. We have a business to run, but its run by people, not machines” [59]
	“I have an employee who has gone through a lot in his life, and got CBT treatment for depression. Based on this experience, he has very good skills to cope with organizational changes and stress compared to my other employees. In this way, he is a resource” [45]
	Knowledge, skills, and experience
	Barriers:
	Lack of knowledge about ABI or mental illness and its impact on employee’s work ability	[Stroke survivor employees] “Such knowledge was however asked for by the employers, as they felt uncertain about their levels of “medical” knowledge and how this affected their responsibility as an employer” (Author interpretation) [42]
	Lack of knowledge and skill regarding supportive strategies for RTW and work retention	“They did not know how best to support their employee, or the extent to which the mental health problem impacted on work ability, social context, and productivity” (Author interpretation) [31]
		[ABI survivor employees] “Patients and employers both noted that line managers’ lack of knowledge of sick leave, and company reorganization, were barriers to RTW” (Author interpretation) [13]
		[Employees with mental illness] “Intervention time was an issue that caused uncertainty. Employers did not know how to determine the necessary support period” [31]
	Challenging dealing with situations arising during RTW process and beyond (e.g., recognising when employee unwell)	“Some employers observed no obvious effects from mental illness on how their employees performed their jobs, although some of those same employees reported experiencing negative effects. It seemed that the effects the employee noticed (for example not being as productive) were not always outwardly observable” (Author interpretation) [62]
	Facilitators:
	Knowledge of depression facilitated communication with employee	“Knowledge of depression provides opportunities to take depressive symptoms into account in the communication with employees with depression. Accurate oral and written information is applied to meet depressive symptoms that make it difficult to remember and concentrate” (Author interpretation) [61]
	Benefitted from advice and information from health professionals	[TBI survivor employees] “They welcomed practical advice in planning a phased RTW (e.g. a RTW timetable), guidance about which work tasks to begin with and how to upgrade tasks, and advice on legal requirements regarding driving” (Author interpretation) [55]
	Previous experiences useful for understanding and handling RTW challenges	[Stroke survivor employees] “The participants described how they tried to use previous experiences from both work and private life to handle the challenges with which they were confronted. They emphasised the usefulness of having other experiences like supporting persons with other diagnoses and other difficulties in returning to work as well as one’s own experience of long-term sick leave. These insights contributed to increased awareness about the complexity in the process of RTW and the importance of having sufficient time” (Author interpretation) [46]
		[Employees with mental illness] “ . . . I have a lot of empathy for what she’s been through, and I’ve spoken to her about some of that from my own experiences at different times, I think that has definitely helped.” [57]
	Work retention facilitated by employers being effective leaders and having links with local services	[Employees with mental illness] “Conflicts in the workplace were also cited as a potential cause of stress, and that conflicts needed to be dealt with quickly by the employer to prevent negative effects” (Author interpretation) [31]
		“Several social firms had links with local mental health services, liaising with services to support employees if their mental health became a cause for concern” (Author interpretation) [54]
Provision of work accommodations	(No sub-theme)
	Barriers:
	Work accommodations not always possible due to impact on co-workers	[Employees with depression, adjustment disorder or anxiety] “Sometimes the doctor thinks, yes, it’s a good idea to make some small adjustments, but that’s not so easy because it affects co-workers . . . ” [43]
	Employers in small- and medium-sized organisations restricted by financial aspects of work accommodations	“With a smaller employer it is harder to offer light duty. Most of the time, a small business employer can’t wait for the worker to recover from a TBI injury. Recovery in those cases, from my experience, is often 6 to 12 months. In order for a small business to survive they can’t wait that long before filling that position” [59]
		[Employees with mental illness] “ . . . it’s right that they’re supported, but it’s just really hard. It has a big impact on other colleagues and a big impact on the business reputation and growth.” [56]
	Employers in large organisations restricted by negative attitudes of senior management towards accommodations	[Employees with episodic disabilities, e.g., depression, anxiety] “ . . . HR participants and DMs reported that their efforts to build awareness, increase training, and provide accommodations for workers with episodic disabilities were seen by their senior management as expensive and time consuming and as not contributing to the bottom-line of the organization” (Author interpretation) [48]
	Lacked autonomy, time and availability to provide support for employees with mental illness	[Employees with mental illness] “ . . . supposed to be at the manager’s discretion but it’s not really, it’s . . . I can decide I want to apply discretion and then I have to send a bid with the case up to my senior managers for them to go “yes that’s ok.”’[57]
		[Employees with mental illness] “Several supervisors referred to their workload which was increasing continuously, with large teams to manage in a difficult work context marked by the lack of human and financial resources. They did not have time to follow up on absent workers and only dealt with the most urgent files” [52]
		[Employees with mental illness] “We have a well written return-to-work policy and action plan for this; the problem is that we do not have the time to follow things through” [47]
	Organisational restructuring during employee absence created challenges in providing support (e.g., ensuring appropriate work role)	(ABI survivor employee) “As a result of the reorganization, he was . . . placed in the administration department . . . Well, if there’s one job . . . he’s not good at, that’s administration” [13]
		(TBI survivor employee) “... we haven’t really had any vacant positions where we can use a handicapped person... the way our plant is structured, that could pose a problem for them.” [64]
	Providing extra support was burdensome on employers	[Employees with mental illness] “If you delegate something to them, you got to hover over them to get it done [. . .] so it can place weight on you also” [60]
Influence from stakeholders	Employees
	Barriers:
	Employees hindered their own RTW through their attitudes and behaviours	[TBI survivor] “Carl reportedly did not attempt to compensate for his poor memory and he may have been unaware of some of his problems or the extent of them” (Author interpretation) [58]
		[ABI survivor employees] “Employers noted that if the patient was too driven, for example by the need to maintain financial security, the resulting stress might threaten successful RTW” (Author interpretation) [13]
	Facilitators:
	Employees with mental illness who retained working roles had certain qualities	“Employers often talked in terms of the qualities that their employee brought to their organisation, rather than benefits. These qualities included insight, respect (commanded for their views as service users with lived experience), knowledge and honesty around their mental illness, creativity, confidence, professionalism, trustworthiness, supportiveness, resilience and credibility” (Author interpretation) [62]
	Helpful when employees used lived experience of mental illness to enhance job performance	“John’s view is that Charlotte^*^’s experience of mental illness adds value to her work, in terms of her ability to engage, relate and validate people’s experiences, making her a better counsellor. As a result, she has a very high retention rate” (Author interpretation) [62]
	RTW of employees with ABIs facilitated by their retained pre-injury orientation and communication skills	“Patients and employers identified several factors facilitating RTW, such as the patient’s drive. Patients and employers agreed that good job performance prior to ABI facilitated RTW” (Author interpretation) [13]
	**Other stakeholders (e.g., family, insurance agencies, health and social care professionals, employers and their superiors, Human Resources/Occupational**
	**Health staff)**
	Barriers:
	Lack of communication across stakeholders caused issues in RTW process, including lack of defined roles	[Employees with depression, adjustment disorder or anxiety] “Lack of clarity between the primary health care services and the OHS regarding the medical and RTW-support available could also add to conflicts and the risk of employees slipping through the net. Therefore, it was important to clarify roles and responsibilities through an open dialogue between the different stakeholders” (Author interpretation) [43]
	Employers’ supportive practices and RTW planning restricted when stakeholders try to enforce their different agendas	[Employees with depression] “ . . . employers’ supportive practices are challenged by the different agendas of the vocational rehabilitation stakeholders poisoning the opportunities to provide support” (Author interpretation) [61]
	Health professionals caused issues during the RTW process (e.g., made demands without understanding situation or job requirements)	[Employees with mental illness] “Employers also described often meeting with rehabilitation professionals who were demanding without any understanding for their situation and specific job requirements” (Author interpretation) [45]
	Family and friends put pressure on- or claimed time of employees	[ABI survivor employees] “Patients and employers mentioned pressures at the patient’s home or people claiming a patient’s time as barriers to RTW” (Author interpretation) [13]
	Facilitators:
	Communication across stakeholders within and across organisations useful for planning and providing support for employees’ RTW	[Stroke survivor employees] “ . . . communication with the Swedish Social Insurance Agency was smooth and allowed for more concrete strategies to be developed to handle work demands and to identify appropriate work tasks in relation to the individuals’ actual resources” (Author interpretation) [46]
	Family support at home facilitated monitoring and adjustment of employees’ working roles and hours following ABIs	“Both patients and employers underlined the importance of support from the partner, whose observation of the patient’s functioning at home helped to reset goals during the RTW-process” (Author interpretation) [13]

**Table 4:** Factors influencing employers’ support

Stakeholder /systems (based on the systems defined in the Sherbrooke Model [43])	Barriers			Facilitators		
ABI literature only	Mental illness literature only	Across ABI and mental illness literature	ABI literature only	Mental illness literature only	Across ABI and mental illness literature
Employer		Considered depression to be employee’s private issue [52, 61] Support for RTW not considered worthwhile investment [61, 62] Large workloads, lack of autonomy, and time constraints hindered support to employee [47, 50, 57]	Lack of knowledge about ABI/mental illness and impact on work ability [31, 42] Lack of knowledge/skills for supportive strategies for RTW and retention (including dealing with unexpected issues) [31, 45-47, 50, 56, 57, 60, 62, 63]		Relevant knowledge about depression potentially facilitated planning of communication and workplace environments [61] Open, calm, and non-judgmental communication with employee [62]	Employees with ABI or mental illness still seen as valuable for organisation [13, 45, 54, 56, 59, 62] Employers’ previous experiences of ABI/mental illness from personal and work life [31, 46, 47, 57, 62] Knowledge and skills for increasing employees’ confidence [62, 63] Effective leadership skills [31, 59]
Employee with ABI or mental illness	Would not employ compensatory strategies to facilitate work participation [58]	Uncompromising with accommodations [50]	Did not disclose diagnosis or work-related challenges [48, 53, 61] Not aware of residual limitations or work-related challenges [31, 48, 58, 59] Too driven/highly motivated, could lead to pressure and stress, threaten or hinder RTW [13, 50, 53]	Communicated their limitations [13] Had retained necessary skills for work performance (e.g., team working) and good pre-injury job performance [13, 53, 58].	Disclosed diagnosis to employer [31, 43] Had certain personal qualities, e.g., resilience, good work ethic [43, 44, 61, 62] [Irvine]
Workplace	Organisational re-structuring limited or prevented availability of suitable, alternative job roles [13, 63, 64]	Employers not permitted to have information or be involved in supporting RTW of employee [31, 50] Senior management saw accommodations as being expensive, time-consuming and unbeneficial [48] Lack of defined roles/responsibilities across supervisors and OH staff, and pressure from superiors to control absences [52]	Potential or actual impact of accommodations on co-workers [31, 48, 53, 56, 61, 63] Accommodations not possible due to financial restrictions [59, 61, 63] Lack of HR support mean extra responsibility for employers [53, 56]		Advice from HR and OH staff on legal obligations, management of performance issues, and solutions to facilitate RTW [31, 50, 52, 62, 63].
Healthcare		Hindered contact between employer and employee, and provided insufficient support [43, 45]	Lack of-, or inadequate information about employee [42, 43, 45]	Information gained by requesting employee to obtain it in writing [53] Advice and information from health professionals regarding employee and aspects of RTW process [46, 53, 55, 59]
Insurance/legislative			Insurance agents applied pressure for RTW to happen quickly [46, 50]
Culture/politics	Family and friends put pressure on- or claimed time of ABI survivor employee [13]	Social workers applied pressure for RTW to happen quickly [61]			Support from Swedish Social Insurance Agency, social workers, or public employment services in improving employer confidence [45], and developing strategies to support employee [46]
Across different stakeholders in different systems (e.g., Human Resources and Occupational Health personnel, insurance agents, social worker, Swedish Social Insurance Agency)		Lack of communication across stakeholders [48, 50] Lack of defined stakeholder roles during RTW/retention [43, 47, 52]	Different stakeholders had different agendas, tried to impose decisions [46, 49, 50, 61] (specific examples given elsewhere in table)	Family supported re-setting of goals, or helped with work responsibilities of employee [13, 63]	Workplace links with local sources of mental health support [54, 62]	Employers supported through communications with other stakeholders in managing and planning RTW process [31, 45-47, 49, 51-53, 56, 59, 61-63] (specific examples given elsewhere in table)

**Table 5:** Contextual characteristics reported in study data

Theme	Sub-theme	Contextual characteristics	Associated barrier or facilitator during employee’s RTW or job retention period
Awareness of condition/illness and support needs	Disclosure of condition/illness or support needs	Cultural taboo associated with depression	Employees in Danish [61] and Canadian studies [48] did not disclose depression diagnosis
		Uncertain economic climate within organisation	Stroke survivors did not ask employer for help when needed [53] (study authors felt this was due to a perceived redundancy risk)
	Access to information about employee	Policies and procedures in workplace and healthcare settings	Insufficient information about employee (with ABI or mental illness) to enable employer support [42, 43, 45, 48, 50, 53]
Provision of work accommodations		Organisation size	Small and medium-sized organisations financially restricted in providing accommodations for employees with ABIs or mental illness [56, 59, 61, 63]
			Large organisations: support for employees with mental illness restricted by productivity and absence objectives, and negative attitudes of senior management [48, 52]. Lack of clear guidelines and defined roles caused confusion among supervisors and OH staff across departments [52].
		Organisational re-structuring	Limited or no availability of suitable, alternative roles for employees with ABIs [13, 63, 64]
		Availability of HR or OH support	Lack of support meant extra responsibilities for employer providing support to stroke survivors [53] or employees with mental illness [56]
			Employers received advice from HR staff on managing performance issues in TBI survivors [63], and their legal obligations to employees with mental illness [62]. OH staff facilitated sustainable solutions for employees with mental illness [31]; and signposted employers to psychiatrists not accessible in public health networks [52].
Influence from stakeholders	Other stakeholders	Involvement of insurance agents, social workers, Swedish Social Insurance Agency, or public employment services	Pressure from social workers or insurance agents for employee with ABI or mental illness to RTW quickly [46, 50, 61]
			Support from social workers, Swedish Social Insurance Agency, or public employment services for employer to help with their confidence for supporting employees with mental illness [45], or specific strategies to support employees with ABIs [46]

#### 3.2.1 Awareness of condition/illness and support needs

3.2.1.1 Disclosure of condition/illness or support needs

Across the ABI and mental health literature, employers were not always aware of an employee’s diagnosis or their support needs, and this was due to a lack of communication from the employee themselves. In Danish [61] and Canadian [48] studies, employees reportedly described depression to employers as something else, due to cultural taboo associated with depression. In studies conducted in New Zealand [31] and Sweden [43], where employees had disclosed their mental illness it led to better understanding and supportive action from their employers.

In a UK-based study, stroke-survivor employees had reportedly not asked for help from employers; the authors suggested this was linked to an uncertain economic climate, and the employee’s belief they may be at greater redundancy risk [53]. Employees with ABIs or mental illness were not always aware of their residual limitations and work-related challenges [31, 48, 58, 59]. In one study, where employees with ABIs had communicated their limitations, it led to more realistic expectations and facilitated their RTW [13].

3.2.1.2 Access to information about employee

Employers also experienced barriers accessing information about an employee’s condition/illness. In two Canadian studies [48, 50], employers were omitted from disability support and RTW planning for employees with mental illness; and this information was deemed necessary for employers’ provision of support [31, 50]. Across ABI and mental health literature, employers in Sweden and the UK reported a lack of- or inadequate information from health professionals [42, 43, 45], and costs when obtaining reports [53]. According to the authors, consent and confidentiality issues and faulty systems were partly to blame for challenges accessing information to inform RTW decisions [53].

Employers of stroke survivors in a UK study had overcome these issues by requesting the employee obtain it in writing from health professionals [53]. In the USA, information from doctors increased understanding of a TBI survivor employee’s abilities and informed planning of the RTW [59].

#### 3.2.2 Employers’ attitudes, knowledge, skills, and experience

3.2.2.1 Attitudes towards condition/illness and support needed

Another barrier was that employers’ willingness to support depended on whether they saw an employee’s mental illness as a workplace- or personal issue [52, 61]; and whether they saw provision of support as a worthwhile investment [61, 62]. In Canada, employees deemed as having personal issues were reportedly scrutinised and pressured to RTW [52].

Across various countries, employers believed employees with mental illness or ABIs were valuable for their organisations [13, 45, 54, 56, 59, 62], and this facilitated their willingness to support these individuals to return to- and stay in work. One example included an employer covering more work to give the employee extra sick leave [62].

3.2.2.2 Knowledge, skills and experience

Across several studies in various countries, employers’ support was hindered by their lack of knowledge about ABI or mental illness, and its impact on work ability [31, 42, 45, 46, 50, 61]. Where employers lacked knowledge of cognitive problems associated with ABIs, authors felt it led to misinterpretations [55] and inadequate workplace environments [59]. In Sweden, where employers lacked knowledge of mental illness, they experienced conflict and uncertainty supporting employees to RTW [45]. Authors stated that where employers had relevant knowledge, it potentially improved their attitudes towards depression, and facilitated planning of communication and workplace environments [61].

Across several countries, employers’ support was also hindered by their lack of knowledge and/or skill regarding supportive strategies for RTW and retention of employees with ABI and/or mental illness [31, 45–47, 50, 56, 57, 60, 62]. This included a lack of knowledge regarding legal obligations and responsibilities [53, 62], the appropriate strategy to use for contacting an employee early on [47, 52], ways of determining a support period [31], understanding what to expect from employees [31, 64], and knowledge about sick leave policies and company reorganisation [13, 48]. In the ABI literature, Swedish [46] and UK-based studies [53, 55] reported that employers’ support was facilitated by advice and information from health professionals regarding work modifications, legal requirements on driving, dealing with consequences of TBI/stroke, grading of tasks, and planning and monitoring a phased RTW. Employers’ previous experiences from personal and work life (especially dealing with mental illness) also facilitated understanding and handling of RTW challenges [31, 46, 47, 57, 62].

Employers felt having the skills to engage in open, calm, and non-judgmental communication enabled them to learn about the employee, their mental illness, and potential needs [62]. Knowledge and skills relating to increasing employees’ confidence (e.g., through work participation and positive reinforcement) were also considered important, whether employees had an ABI [63] or mental illness [62].

Across ABI and mental health literature, other barriers experienced by employers related to skills for dealing with unexpected issues, such as: recognising when an employee was unwell or struggling [62]; supporting an employee with cognitive difficulties [63]; managing employees’ performance/capability issues and unrealistic expectations [56, 63]; and understanding employees’ personality changes and behaviours [63]. Employers also found it challenging to support TBI survivor employees [63] and employees with mental illness [50] to accept they would not be performing at pre-injury/illness levels when they returned to work. Authors stated that TBI survivors with high motivation and drive to return to previous roles were challenging to manage from a performance perspective, due to ongoing difficulties and their persistence [63]. Some of these employees reportedly developed anxiety and depression, and employers struggled to find them meaningful, appropriate duties.

Employer skills in effective leadership (e.g., managing work conflicts early and planning to review the RTW process with others) reportedly facilitated retention of employees with TBIs [59] or mental illness [31]. Retention of employees with mental illness in the UK and New Zealand was also facilitated through organisations having links with local sources of support [54, 62].

#### 3.2.3 Provision of work accommodations

Across the ABI and mental health literature, the potential or actual impact on co-workers could act as a barrier to employers providing work accommodations. The absence of an employee, for example, sometimes meant co-workers were required to work harder for lengthy time periods, sometimes experiencing frustration, stress, distress, and anxiety [48, 56, 61, 63]. Co-workers could also experience jealousy if expected to provide long-term support to an employee with these conditions, or if they saw accommodations provided for the employee [31, 48, 53]. Some employers reported challenges supporting employees with mental illness or TBIs due to conflict between meeting employees’ needs and meeting co-workers’ needs [56, 61], or protecting co-workers from potential harm [53, 63].

Other barriers specific to organisational contexts related to financial status, organisational objectives, inadequate guidelines or training, employers’ own workloads, and organisational re-structuring. Employers within medium- and small-sized organisations were restricted by financial aspects of work accommodations [56, 59, 61, 63]. For example, in small organisations provision of accommodations (e.g., lighter duties) to employees with TBI or mental illness was not sustainable because it negatively impacted productivity, business reputation and growth, and could even threaten survival of the business [56, 59]. In large Canadian organisations, accommodations for employees with depression were restricted by productivity and absence objectives [52] and senior management attitudes (e.g., seeing work accommodations as costly and unbeneficial) [48]. In other studies, employers struggled to provide support due to lack of autonomy (i.e., needing to have changes approved by senior management) [57], and time and large workloads [47, 50]; and extra support for employees with mental illness [56, 60] and TBIs [63] had proven burdensome. Employers of ABI survivors [53] or mental illness [56] in the UK had taken on extra responsibility due to unavailability of HR support. In the ABI literature, organisational re-structuring limited or prevented availability of suitable, alternative work roles for employees [13, 63, 64].

#### 3.2.4 Influence from stakeholders

3.2.4.1Employees

Employees’ attitudes, behavi-ours, and personal qualities could hinder or facilitate the success of employers’ support for their RTW or job retention. Authors reported an ABI survivor did not attempt to use compensatory strategies for his memory to aid job performance, potentially because he was unaware he had memory problems [58]. Others reported the following issues among employees with mental illness: “overdoing” it following RTW; or being closed-minded and uncompromising with proposed work accommodations [50]. Similarly, if employees with ABIs were too motivated, it could result in stress and pressure and threaten or hinder their RTW [13, 53]. In a UK-based study, authors’ suggested reasons for RTW motivation among stroke survivors included financial insecurity, and guilt relating to perceived loss of status and burden on co-workers [53].

Employers’ retainment of employees with mental illness in working roles was facilitated by these employees having certain qualities, including: knowledge and honesty around their illness and work ability; creativity; trustworthiness; resilience; professionalism; a good work ethic; good communication skills; and optimism [43, 44, 56, 61, 62]. In other studies, employers considered it helpful when ABI survivors’ had retained pre-injury orientation and communication skills; team-working skills; and good pre-injury job performance [13, 53, 58].

3.2.4.2Other stakeholders

Employers’ RTW/retention support was also influenced by other stakeholders involved, including health and social care professionals, employers and their superiors, HR/OH staff, government authorities, insurance agents, and an employee’s family and friends.

In the mental health literature, authors reported that lack of communication across stakeholders led to frustration among workplace actors [48], and delays in the RTW process [50]. At times, there was also lack of clarity over different stakeholders’ roles/responsibilities and support available [43, 47], and in a Swedish study sometimes this meant no one took responsibility, leaving the employee to manage their own RTW [47].

Employers’ support for people with mental illness or ABIs was also restricted when different stakeholders had different agendas, and each stakeholder tried to make things go their way. For example, authors reported that health professionals in Sweden and Canada hindered contact between the employers and employees [43], and made demands without understanding the situation or job requirements [45]. In Canada [50], Sweden [46] and Denmark [61], insurance agencies and social workers reportedly applied pressure for RTW to happen quickly. In the Netherlands, employers and ABI survivors described how family and friends placing pressure on- or claiming time of employees could be a hindrance [13]. Imposition of other stakeholders’ agendas and lack of defined roles/responsibilities could also happen within an organisation, and hinder RTW or retention support. In a large Canadian organisation, government authorities and senior management pressured OH staff and supervisors to control absences and reduce disability insurance costs [52]. The juxtaposition of wanting to support employees with mental illness versus controlling absences, combined with a lack of clear guidelines, meant there were contradictory practices and confusion among supervisors and OH officers in different departments. Sometimes supervisors did very little to support because they saw prevention and management of absences as being the role of OH and HR departments.

Across the ABI and mental health literature, communication across stakeholders within and outside organisations facilitated employers’ RTW and retention support [31, 45–47, 49, 51–53, 56, 59, 61–63]. For example, communication with the Swedish Social Insurance Agency and social workers supported development of task identification and workload management strategies for stroke survivors [46]. In another Swedish study, support from a public employment service improved employer confidence in meeting and supporting employees with mental illness [45]. Additionally, HR staff advised on working with employees with mental illness in Canada [50], managing performance issues in TBI survivors in Australia [63], and legal obligations regarding sick leave and time off for appointments due to mental illness in New Zealand [62]. Communication with OH personnel enabled sustainable solutions for employees with mental illness in Sweden [31]; and signposting to psychiatrists not accessible in public health networks in Canada [52].

In the ABI literature, family members’ observations of employees at home in Australia revealed to employers whether they were coping with increasing working hours and responsibilities [63]. In the Netherlands such observations aided resetting of RTW goals [13].

## 4 Discussion

This review focused on influential factors and surrounding contexts that hindered or facilitated employers’ support for people with ABIs and/or mental illness to return to- and stay in work. Synthesis findings showed that employers’ support was influenced by their awareness/knowledge of- and attitudes towards the employee’s condition/illness; their skills and experience in providing RTW/retention support; factors related to provision of work accommodations; and influence from other stakeholders. Contextual characteristics surrounding influential factors related to organisational characteristics (e.g., organisation size and resources), cultural taboo associated with depression, and involvement of certain stakeholders (e.g., insurance agents). No studies relating to employees with ABI and co-morbid mental illness were identified, so the review data related only to those with singular morbidities (i.e., ABI or mental illness). Nevertheless, findings showed that the RTW process for this population sub-group is potentially more complex. Employers may experience combinations of issues identified only in the ABI literature (e.g., employee’s unwillingness to employ compensatory strategies) or mental illness literature (e.g., employer considering depression a private issue). At the same time, the issues experienced across these population sub-groups may have a compounding affect in instances where an employee has ABI and co-morbid mental illness. Employers may experience greater issues having sufficient knowledge of ABI and mental illness, and in knowing how these uniquely impact the employee and interact to influence their work ability skills. Such employers may also be required to liaise with a greater number of stakeholders with different agendas across different services and systems, and potentially require greater skill in navigating the RTW process (e.g., considering a greater array of factors and how these may impact all involved). The findings reported across the ABI and mental illness literature, and implications relating to employers’ needs, are discussed hereafter.

To begin with, employers reported that employees did not disclose relevant information (e.g., diagnosis, residual limitations); and this was compounded by contextual factors like faulty information sharing systems, and workplace and health system policies regarding consent and confidentiality. The importance of selective information sharing to enable work accommodations has been recognised [67, 68]. For example, a decision support tool has been developed to support people with mental illness with disclosure to employers [69]. In a randomised controlled trial, the tool was statistically significantly effective in reducing decisional conflict, and at 3-months follow-up a greater proportion of the intervention group (*n* = 40) had moved into paid or voluntary employment (15% increase), compared with the control group (*n* = 39) (8% increase) [70]. The authors admit that sample sizes were small, and the tool requires further testing; nevertheless it highlights the potential usefulness of such a tool. Currently, no such tool exists for ABI survivors; though some of the previously mentioned tool’s mechanisms of action [69] (i.e., considering the individual’s needs and values, clarifying pros and cons of disclosure in their situation) correspond with important disclosure decision-making elements reported by ABI survivors [67]. Further research is needed to develop and test a disclosure decision aid usable by ABI survivors. Such an aid may be especially useful among ABI survivors with co-morbid mental illness, given the additional contextual characteristics that may influence disclosure of their limitations or diagnosis (e.g., cultural taboo associated with depression). Additionally, a lack of training for health professionals and services to meet the needs of ABI survivors with co-morbid mental illness has been reported [4]. Different services (including those outside of health and workplace systems) may not be integrated or communicate with one another, making it more complicated and laboursome obtaining information on the employee’s work abilities and rehabilitative prognosis. Employers in these instances may benefit from support from a coordinator in vocational rehabilitation with specialist knowledge of this population, e.g., to advise on communication strategies to facilitate disclosure, assess the ABI survivor’s work abilities, and collate information and advice from different stakeholders regarding the ABI survivor’s work participation and available resources. In the current review, employers found it helpful when an employee disclosed their mental illness diagnosis, and when they were given advice and information from stakeholders regarding the ABI survivor’s work abilities and RTW process.

Across several countries employers lacked knowledge of ABI or mental illness, and knowledge and skills relating to supportive actions. For example, employers struggled to support ABI survivors and employees with mental illness to accept that they may not perform at pre-injury or pre-illness levels when they returned to work. This seemed especially important among ABI survivors, because some of those experiencing difficulty accepting the changes subsequently developed co-morbid mental illness (i.e., anxiety, depression) [63]. Difficulty accepting an ABI and its consequences has been reported as a major RTW barrier by ABI survivors elsewhere [20]. Trialling a working role on a short-term basis (i.e., a work trial) can prevent confrontation of limitations for ABI survivors [63, 71], and has been cited by employers of TBI survivors as being helpful [63]. In order to provide a work trial however, employers would need to know it was the appropriate action to undertake with employees in that situation. This review thus highlights that employers may benefit from education on supportive strategies, including ways of reducing the risk of ABI survivors developing co-morbid mental illness.

Employers of ABI survivors or people with mental illness also benefitted from advice from various stakeholders (e.g., health professionals, social workers, HR and OH staff) regarding their confidence and responsibilities, and practical elements needed in planning, conducting, and monitoring a phased RTW. There is strong evidence that effective, patient-focused RTW interventions for ABI survivors combine work-directed components (e.g., task adaptation) with education/coaching (e.g., emotional support) [72]. However, it seems as though all of these interventions required support from a specialist coordinator, and not all ABI survivors or their employers have this support. Where a specialist coordinator is not available, ABI survivors (with or without diagnosed co-morbid mental illness) may benefit from an accessible, self-guided resource to use with employers to educate them on planning, conducting, and monitoring a sustainable RTW. It may prove useful for the resource to include signposting to local sources of support, as support links facilitated retention of employees with mental illness in the current review [62].

Among included studies, restriction of work accommodations was generally due to employers’ concerns about the actual or potential impact of accommodations on co-workers of the employee with ABI or mental illness. Others have reported similar findings; with some employers even refusing to provide accommodations, believing it to be discriminatory to non-disabled employees [73]. Elsewhere, ABI survivors with co-morbid mental illness have reported social stigma from others and poor attitudes and insight relating to disabilities [4]. The importance of support from employers and co-workers for ensuring RTW and retention is well-recognised across ABI and mental health literature [16–18, 22]. RTW models and policies should include consideration of social relations between workplace actors, and involve co-workers in RTW plans [74]. Additionally, in studies mostly including large organisations, negative attitudes of senior management (e.g., focusing on absence/productivity objectives and costs of accommodations) restricted support for employees with mental illness. It has been suggested that education for all stakeholders regarding employment rights and indicators of stigma and discrimination is needed, as well as support for employees to self-advocate in the workplace [67]. The effectiveness of anti-stigma interventions for mental illness in workplaces is inconclusive [75], and evidence is non-existent regarding ABIs. However, commonly suggested anti-stigma strategies include education from people with lived experience of the condition/illness and awareness campaigns [76, 77].

Across most studies, it was unclear whether contextual characteristics (e.g., country, occupation type, organisational size and industry) may have directly influenced employer support, because a breakdown of results across different types of organisations, etc, were not always provided. However, in some studies employers in small and medium-sized organisations struggled to provide accommodations due to financial implications [56, 59, 61, 63]. Elsewhere, statistically significant positive associations between organisation size and RTW outcomes among stroke survivors (i.e., odds of RTW [78], shorter time to RTW [79]) have been reported. These associations may be due to larger organisations having more experience and resources to support RTW and job retention, though such differences may be mitigated in countries where RTW is externally subsidised [78]. Additionally, one study in this review highlighted the pressure within large organisations to maintain productivity and reduce absence rates, and it is likely this would lead to a quicker RTW among sick-listed employees. Given the small amount of data concerning contextual characteristics, further research is warranted to explore the influence of these characteristics on employers’ RTW and retention support for people with ABIs and/or mental illness. Furthermore, the issue of co-morbid mental illness and economical inactivity (i.e., people who are not working nor looking for work) is a growing issue. Since the beginning of the COVID-19 pandemic, the number of people in the UK economically inactive due to long-term sickness, has risen by over 400,000 to a total exceeding 2.5 million [80]. In the first quarter of 2023, more than one million of these reported having depression, anxiety, or nerves as a health condition secondary to a main condition. Greater understanding of the influence of contextual characteristics, such as organisation size, type, and industry, may reveal changes that could be made at multiple levels to support people with ABI and co-morbid mental illness, and reduce economic inactivity rates.

Another limitation of the included studies was that they did not report on the cultural diversity or immigrant statuses of employers and/or their employees. Thus, it is unclear whether these socio-demographic characteristics could have influenced employers’ support (or employees’ reception of support). It is recommended that future research explore this further. Increasing understanding may ensure that future work to improve employers’ support does not neglect the needs of those who are underserved, or have protected characteristics.

### 4.1 Strengths and limitations of the review

In order to maximise identification of relevant studies, a broad search strategy was used across various relevant databases. The RETREAT framework [39] was employed to ensure the choice of synthesis methodology was appropriate.

During preliminary scoping searches, potentially eligible studies involving multiple populations, e.g., those with mental illness or musculoskeletal injuries, did not always report a breakdown of their findings per population group. To ensure relevancy of findings, these particular studies were required to report 50% or more of employer participants as having previous experience supporting employees with ABIs or mental illness to return to- or stay in work. Upon reflection, a better approach may have been to exclude these papers, to avoid including small amounts of data potentially relating to other conditions or injuries.

Given the paucity of the evidence base, it was not possible to limit the countries in which the included studies were based. The included studies therefore varied in their social assurance systems, health systems, legislation, and legal requirements for employers’ RTW and retention support. For example, involvement of the Swedish Social Insurance Agency was specific to Swedish studies. Thus, the transferability of some findings is specific to certain countries and may not apply to others with different systems.

Due to time constraints, only one reviewer completed the screening of titles and abstracts, and the first stage of the thematic synthesis. However, multiple reviewers were given access to the coded data and involved in the second and third stages of the synthesis. An English language restriction was used; deemed necessary due to the language skills of the reviewers involved and time constraints. Despite this, studies from various non-English speaking countries were included.

## 5 Conclusion

Employers’ support for ABI survivors or individuals with mental illness to return to- and stay in work is influenced by various factors, involving different stakeholders across different systems. ABI survivors (with or without co-morbid mental illness) may benefit from an accessible, self-guided resource to use with employers to guide them on planning, conducting and monitoring a sustainable RTW. The RTW process may also be facilitated by involvement of a specialist coordinator, provision and use of a disclosure decision aid, education for employers on supportive strategies, consideration of co-workers in RTW policies and planning, deployment of anti-stigma strategies, and support for employee self-advocacy. Further research is needed to investigate employers’ knowledge requirements, and explore the influence of other stakeholders, socio-demographic characteristics, and contextual factors on employers’ RTW/retention support for ABI survivors with co-morbid mental illness.

## Ethical approval

This study is a systematic review, and is therefore exempt from Institutional Review Board approval.

## Informed consent

This study is a systematic review and reports published data, therefore informed consent was not required.

## Data availability

The data that support the findings of this review are openly available in Nottingham Research Data Repository at http://doi.org/10.17639/nott.7262.

## Conflict of interest

The authors declare that they have no conflict of interest.
